# Genotype distribution and identification of rare variants in α/β-thalassemia in chengdu: including the first report of HBA1:c.300+48C > G

**DOI:** 10.3389/fgene.2026.1899397

**Published:** 2026-10-16

**Authors:** Xiaohua Liu, Hongfei Du, Linyan Zhang, Yang Yu, Guixiang Wang, Xing He, Huacui Huang, Ying Xu

**Affiliations:** 1 Department of Medical Laboratory, Xindu District People’s Hospital of Chengdu, Chengdu, Sichuan, China; 2 Department of Clinical Laboratory, The First Affiliated Hospital of Chengdu Medical College, Chengdu, Sichuan, China

**Keywords:** gene variant, genotype, HBA1:c.300+48C > G, α-thalassemia, β-thalassemia

## Abstract

**Objective:**

Thalassemia is among the most common inherited diseases worldwide. We aimed to analyze the genotype and frequency distribution of thalassemia in a general hospital in Chengdu and provide a reference for genetic counseling and prenatal diagnosis.

**Methods:**

A total of 6,357 subjects referred to Xindu District People’s Hospital of Chengdu for thalassemia screening between June 2021 and December 2025 were collected. Thalassemia genotypes were tested using polymerase chain reaction (PCR), flow-through hybridization technology and Sanger sequencing analyses.

**Results:**

A total of 793 subjects heterozygous or homozygous for alpha/beta-globin mutations were detected from 6,357 blood samples, including 480 α-thalassemia variants, 291 β-thalassemia variants, and 22 cases with concurrent α and β-thalassemia variants. One novel variant, HBA1:c.300 + 48C > G, and was not detected in the IthaGenes, HbVar, dbSNP, Pubmed, and HGMD databases.

**Conclusion:**

The genotype distribution of thalassemia in a general hospital in Chengdu is complex and heterogeneous. The novel HBA1 variant was identified in a patient with severe anemia; however, its functional impact and clinical significance require further investigation.

## Introduction

Thalassemia is a group of inherited hemolytic anemias caused by an imbalance in globin peptide chain synthesis due to defects in the globin gene, and is one of the most common single-gene inherited diseases worldwide (Tuo et al., 2024; Xian et al., 2022). The geographical distribution of thalassemia exhibits distinct characteristics, particularly in the Mediterranean region, Southeast Asia, and certain areas of China. In China, regions south of the Yangtze River, especially provinces such as Hainan, Guangdong, Guangxi, Fujian, Hunan, Guizhou, Sichuan, and Yunnan, have a higher incidence of thalassemia (Xian et al., 2022; Chen et al., 2022; Wang et al., 2022; Xu et al., 2022). Currently, there is a lack of effective treatments for severe thalassemia. Terefore, carrier screening, prenatal diagnosis, and pre-implantation genetic screening are currently the only measures that can effectively prevent the birth of children with severe thalassemia and improve the quality of life for this population (Shook et al., 2020). The clinical diagnosis of thalassemia is currently often achieved by screening with routine blood tests and hemoglobin electrophoresis, followed by genetic testing for those with abnormal results. Therefore, this study analyzed thalassemia gene screening samples conducted in Xindu District, Chengdu City, from 2021 to 2025, and preliminarily obtained the genotypes and frequency distribution of thalassemia gene variants in this region. Our research findings contribute to enriching the relevant information on thalassemia in this area and provide important evidence for genetic counseling and prenatal diagnosis work in Xindu District of Chengdu.

## Materials and methods

### Research subjects

A total of 6,357 individuals who underwent thalassemia screening at Xindu District People’s Hospital of Chengdu between 2021 and 2025 were included in this study. Screening was conducted as part of routine premarital, prenatal, or general health check-up programs according to local public health policies. The predominance of females reflects the higher proportion of individuals presenting for prenatal screening in our setting. We used the hospital information system to exclude duplicate records based on ID numbers, names, and contact information. All samples were obtained from peripheral venous blood; no amniocentesis or chorionic villus sampling samples were included. All procedures were conducted following the ethics guidelines for research on human subjects. This study was approved by the Ethics Committee of Xindu District People’s Hospital of Chengdu.

### Hematological analysis

Peripheral venous blood (2 mL) was collected in EDTA anticoagulant tubes. Hematological parameters were determined using a BC-6900 automatic hemocytometer (Mindray, Shenzhen, China). Hemoglobin analysis was performed using a capillary electrophoresis analyzer (CAPILLARYS 3 TERA, SEBIA, France).

### Whole blood gDNA extraction

Genomic DNA was extracted from peripheral venous blood using a DNA Rapid Extraction Kit (Tianlong Tech-nology Co., Ltd., Xi’ an, China). DNA concentration andpurity were determined by ultraviolet spectrophotometer (Thermo Fisher Technologies, United States).

### Analysis of routine α/β-thalassemia genotypes

The experiment was conducted in accordance with the instructions of the thalassemia gene detection kit (Guangdong Hybribio Biotech Co. Ltd., Guangdong, China). To identify three common deletional α-thalassemia mutations (--SEA/NC_000016. 9: g. 215400_234700del, -α3.7/NC_000016. 9: g. 223300_227103del, -α4.2/NC_000016. 9: g. 219817_(223755_224074) del) and three common non-deletional α-thalassemia mutations (αCS/*HBA2:c.427T>C*, αQS/*HBA2:c.377T > C*, αWS/*HBA2:c.369C > G*) for α-thalassemia. The 17 common β-thalassemia mutations detected were as follows:

*−28(A > G)*/ *HBB:c.-78A > G, −29(A > G)/ HBB:c.-79A > G, −30(T > C)/ HBB:c.-80T > C, −32(C > A)/ HBB:c.-82C > A,CD41/42(-TTCT)/HBB:c.126_129del,CD43(G > T)/HBB:c.130G > T,CD17(A > T)/HBB:c.52A > T,CD14-15(+ G)/HBB:c.45_46insG, IVS-I-1(G > A/T)/HBB:c.92 + 1G > A or HBB:c.92 + 1G > T,IVS-I-5(G > C)/HBB:c.92 + 5G > C,βE(G > A)/HBB:c.79G > A,IVSII-654(C > T)/HBB:c.316-197C > T, CD71-72(+ A)/HBB:c.216_217insA, CD27-28(+ C)/HBB:c.84_85insC, Cap(-AAAC) or A > C/HBB:c.-11_-8delAAAC, Int (T > G)/ HBB:c.93-21T > G, CD31(-C)/HBB:c.94delC*.

### Sanger sequencing in patients with rare thalassemia

Gene sequencing was performed in cases with negative routine thalassemia testing but presenting with abnormal clinical phenotypes or hemoglobin component analysis (e.g., HbA2), to identify rare mutations. Two sets of primer pairs were used: α1-F: 5′-CTCCGCGCCCAGCCAA -3’; α1-R: 5′-GCA​GAG​AGG​TTC​TAG​CCA-3’; α2-F: 5′-CGC​GCC​AGC​CAA​TGA-3’; α2-R: 5′-GCT​GCA​GAG​AGG​TCC​TG-3’.

We sequenced β globin gene with three pairs of primers: G1 (forward primer B1F: 5′-GGC​TGT​CAT​CAC​TTA​G-3′; reverse primer B1R-2: 5′-TCA​CTC​AGT​GTG​GCA​AA-3′), G2 (forward primer B2F: 5′-CTG​TTA​TGG​GCA​AC-3′; reverse primer B2R: 5′-TAT​TGC​CTT​AAC​CCA​G-3′), and G3 (forward primer B3F: 5′-TCA​TGC​CTC​TTT​CAC-3′; reverse primer B3R: 5′-GTT​TTA​AAT​GCA​CTG​A-3′). Amplification was carried out using a PCR instrument (ABI Veriti® 96-well thermal cycler, Thermo Fisher, United States) with a final primer concentration of 0.5 μmol/L. The PCR products were then sequenced on a sequencer (3500xL Genetic Analyzer, Thermo Fisher, United States).

### Statistical analysis

Microsoft Excel 2007 and GraphPad Prism 5 software were used for processing and analysis, and SPSS 26.0 software was used for statistical analysis. Count data were expressed as number of cases (n) or percentage (%). And differences between the two groups in each hematological index were compared using the Mann-Whitney U test. A p-value < 0.05 was considered statistically significant.

## Results

### Genotype distribution of routine α/β-thalassemia

A total of 793 subjects with confirmed globin gene mutations were identified among 6,357 screened individuals (12.47%), comprising 480 α-globin mutations (60.53%), 291 β-globin mutations, (36.70%), and 22 subjects with confirmed globin gene mutations of coexisting α-and β-globin mutations (2.77%). The mean age of the overall cohort was 28.56 ± 8.25 years, with females accounting for 89.04% of participants. Among subjects with confirmed globin gene mutations, 584 were female (73.64%) and 209 were male (26.36%), with a mean age of 27.20 ± 16.82 years; the majority (69.86%, n = 554) were aged 20–39 years.

Among 480 subjects with confirmed α-globin mutations, *--* ^*SEA*^ */αα* was the most common genotype, Other common genotypes of *α*-thalassemia included *-α* ^*3.7*^ */αα*, *-α* ^*4.2*^ */αα* and *αα* WS*/αα* (Table 1). Among 291 subjects with confirmed β-globin mutations, The most frequent genotypes were *β* ^*CD17(A>T)*^ */β* ^*N*^, *β* ^*CD41-42(-TCTT)*^ */β* ^*N*^ and *β* ^*IVSII−654,*^,collectively representing 81.10% of all β-thalassemia genotypes. Other common genotypes of β-thalassemia included *β* ^*−28(A>G)*^ */β* ^*N*^ (*β* ^*βE(G>A)*^ */β* ^*N*^, and *β* ^*CD71-72(+A)*^ */β* ^*N*^ (18.9%) (Table 1).

**TABLE 1:** Genotype distribution and corresponding phenotypes in subjects with α-thalassemia, β-thalassemia and α/β-globin mutations.

α-thalassemia			β-thalassemia			α/β-thalassemia		
Genotype	Cases (%)	Phenotype	Genotype	Cases (%)	Phenotype	Genotype	Cases (%)	Phenotype
*--SEA/αα*	221 (46.04)	α0/αα	*β* ^*CD17(A>T)*^ */β* ^*N*^	86 (29.55)	β0/βN	*-α3.7/αα,βIVSII-654(C > T)*	5 (22.73)	αα+/αα; β+/βN
*-α3.7/αα*	179 (37.29)	αα+/αα	*β* ^*CD41-42(-TCTT)*^ */β* ^*N*^	80 (27.49)	β0/βN	*-α3.7/αα,βCD17(A > T)*	4 (18.18)	αα+/αα; β0/βN
*-α4.2/αα*	29 (6.04)	αα+/αα	*β* ^*IVSII−* *654*^ *(C>T)/β* ^*N*^	70 (24.05)	β+/βN	*-α3.7/αα,βCD41-42(-TCTT)*	2 (9.09)	αα+/αα; β0/βN
*α WSα/αα*	14 (2.92)	αα+/αα	*β* ^*−* *28* *(A>G)*^ */β* ^*N*^	24 (8.25)	β+/βN	*--SEA/αα,βCD17(A > T)*	1 (4.55)	α0/αα; β0/βN
*--SEA/-α3.7*	12 (2.50)	α0/α+	*β* ^*βE(G>A)*^ */β* ^*N*^	11 (3.78)	β+/βN	*--SEA/αα,βCD41-42(A > T)*	1 (4.55)	α0/αα; β0/βN
*αCSα/αα*	14 (2.92)	αα+/αα	*β* ^*CD71-72(+A)*^ */β* ^*N*^	6 (2.06)	β0/βN	*--SEA/αα,β-29(A > G)*	1 (4.55)	α0/αα; β+/βN
*αQSα/αα*	8 (1.67)	αα+/αα	*β* ^*−* *29* *(A>G)*^ */β* ^*N*^	7 (2.41)	β+/βN	*αCSα/αα,βCD17(A > T)*	1 (4.55)	αα+/αα; β0/βN
*--SEA/-α4.2*	2 (0.42)	α0/α+	*β* ^*CD27-28(-TCTT)/*^ *β* ^*N*^	3 (1.03)	β0/βN	*αQSα/αα,βIVSII-654(C > T)*	1 (4.55)	α+/αα; β+/βN
*-α* ^*3.7*^ */-α* ^*4.2*^	1 (0.21)	α+/α+	*β* ^*Cap(-AAAC)*^ */β* ^*N*^	2 (0.69)	β+/βN	*-α4.2/αα,βCD41-42(-TCTT)*	1 (4.55)	αα+/αα; β0/βN
​	​	​	*β* ^*CD43(G>T)*^ */β* ^*N*^	2 (0.69)	β0/βN	*αCSα/αα,βCD71-72(+ A)*	1 (4.55)	αα+/αα; β0/βN
​	​	​	​	​	​	*--SEA/αCSα,βCD17(A > T)*	1 (4.55)	α0/α+; β0/βN
​	​	​	​	​	​	*α WSα/αα,βCD17(A > T)*	1 (4.55)	αα+/αα; β0/βN
​	​	​	​	​	​	*-α3.7/αα,β-28*	1 (4.55)	αα+/αα; β+/βN
​	​	​	​	​	​	*-α3.7/αwsα,βIVSII-654(C>T)*	1 (4.55)	α+/α+; β+/βN
Total	480 (100)	​	​	291 (100)	​	​	22 (100)	​

22 subjects with confirmed both α-and β-globin mutations. Seventeen genotypes were detected in these subjects, and the most common type was *-α* ^*3.7*^ */αα* combined with *β* ^*IVSII−654(C>T)*^ */β* ^*N*^ (Table 1).

There were significant differences in the basic hematological parameters between patients with α-and β-thalassemia. Patients with β-thalassemia exhibited lower MCV (65.91 ± 6.81 fL vs. 76.11 ± 10.61 fL, P < 0.001) and MCH (20.84 ± 2.32 pg vs. 24.54 ± 4.41 pg, P < 0.001) compared to those with α-thalassemia. MCHC was also slightly lower in the β-thalassemia group (316.18 ± 9.38 g/L vs. 319.44 ± 10.26 g/L, P < 0.001), These findings indicate that MCV and MCH are more reliable discriminators between α- and β-thalassemia phenotypes (Table 2).

**TABLE 2:** The differences of hematological parameters between a-thalassemia and β-thalassemia.

Parameter	a-thalassemia mean ± SD	β-thalassemia mean ± SD	p value
MCV(FL)	76.11 ± 10.61	65.91 ± 6.81	P < 0.001
MCH (pg)	24.54 ± 4.41	20.84 ± 2.32	P < 0.001
MCHC(g/L)	319.44 ± 10.26	316.18 ± 9.38	P < 0.001

MCV, mean corpuscular volume; MCH, mean corpuscular hemoglobin content; MCHC, mean corpuscular hemoglobin concentration.

### Identification of rare α/β-thalassemia genotypes

Identification of rare α/β thalassemia genotypes rare globin gene variants were detected in 10 patients (cases 1–10, Table 3), including 4 patients with rare α-thalassemia (case1-4), 5 patients with rare β-thalassemia (cases 5–9). 1 patients with α-thalassemia combined with rare β -thalassemia (cases 10). In this study, one new pathogenic gene variant of α-thalassemia cases were identified, including case 4: HBA1:c.300 + 48C > G, the variant has not been reported in HbVar, HGMD, and PubMed databases.

**TABLE 3:** Rare globin gene variants identified in subjects with α-thalassemia (n = 4), β-thalassemia (n = 5) and α-thalassemia combined with β -thalassemia (n = 1).

No.	Sex-age	HBA genotype	HBB genotype	Hb (g/L)	MCV (fL)	MCH (pg)	HbA (%)	HbA2 (%)	HbF (%)
Case 1	F-31	HBA2:c.51G > T Compound HBA2:c.300 + 55T > G	β^N^/β^N^	127	101.6	33.4	71.0	2.0	0.0
Case 2	M-65	HBA2:c.301-24delGinsCTCGGCCC	β^N^/β^N^	128	81.3	24.4	97.1	2.2	0.0
Case 3	F-26	HBA1:c.300 + 13C > G	β^N^/β^N^	120	94.4	30.9	96.7	1.3	2.0
Case 4	M-31	HBA1:c.300 + 48C > G	β^N^/β^N^	69	70.1	19.5	97.9	2.1	0
Case5	F-26	αα/αα	HBB:c.68A > C	112	85.9	28.8	54.1	3.1	0.0
Case 6	F-30	αα/αα	HBB:c.198G > T	138	88.7	29.3	94.2	1.5	4.3
Case 7	F-32	αα/αα	HBB:c.*233G > C	104	92.6	29.9	97.1	1.9	1
Case 8	F-29	αα/αα	HBB:c.-81A > C	116	69.9	23.3	94.3	4.6	1.1
Case 9	F-34	αα/αα	NG_000007.3:g.48795_127698del	113	69.9	22.5	82.3	2.4	15.3
Case 10	M-11	NC_000016.10:g.149863_183312del;	HBB:c.246C > A	111	65.7	20.7	97.5	2.5	0

Case 4, Male, 31-year-old, ancestral home in Chengdu with severe anemia. The patient exhibited no jaundice and had no history of blood transfusion. Admitted on 22 December 2023, due to “cough with expectoration, accompanied by fever and fatigue for more than 3 days” Hematological parameters for this patient were measured after recovery from the acute febrile episode, ensuring that the reported values reflect the stable baseline status. Laboratory assessments revealed negative results for routine thalassemia gene tests, and ferritin level was greater than 1,500 ng/mL.The hemoglobin profile was as follows: HbA, 97.9%, HbA2, 2.1%, and Hb F, 0%. Sanger sequencing revealed a novel heterozygous mutation in the a-globin gene HBA1:c.300 + 48C > G (Figure 1).

**FIGURE 1:**
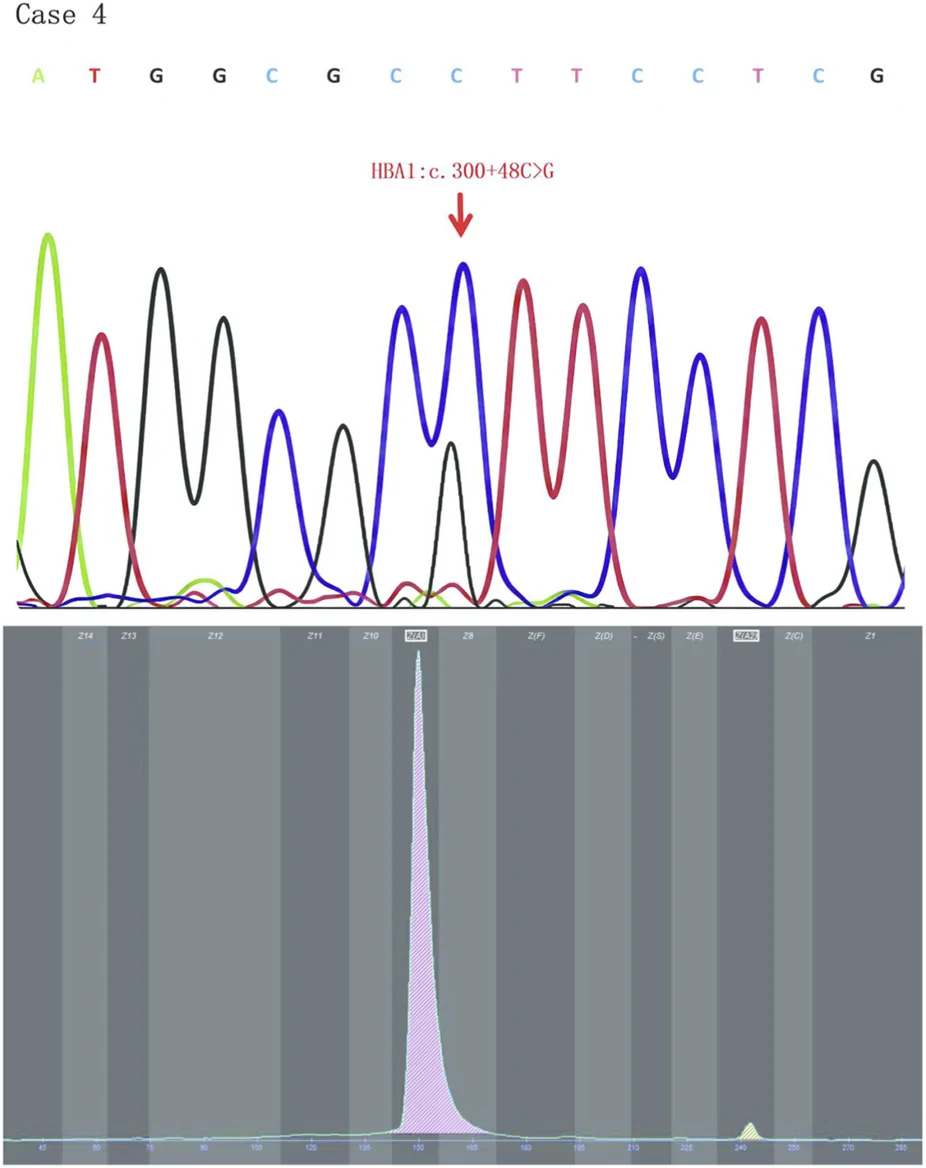
Novel HBA1:c.300 + 48C > G variant in Case 4.

## Discussion

Among the 6,357 samples analyzed, the overall detection rate of thalassemia mutations was 12.47%. Of these cases, α-globin mutations were detected in over 60%, β-globin mutations in approximately 40%, and coinheritance of both α-and β-globin mutations in less than 3%.

In our study, out of the 793 subjects with confirmed globin gene mutations, 584 (73.64%) were female, and 209 (26.36%) were male. Specifically, more than 60% of patients were aged between 20 and 39 years, and most were young or middle-aged women. Because in this study, pregnant women at our hospital undergo routine screening for thalassemia, and if the initial screening is abnormal, routine thalassemia genetic testing is performed. It is very common in clinical practice for pregnant women to undergo thalassemia genetic testing, mainly because it serves as a crucial step in preventing the birth of children with severe thalassemia (Zhang et al., 2026). In addition, due to the loss of menstrual blood in women, compared with men, women are more likely to suffer from anemia and are more inclined to seek medical treatment for testing (Ghazala Nazir et al., 2011; Nair et al., 2022).

In 480 subjects with confirmed α-globin mutations, the most frequent genotype was*--* ^*SEA*^ */αα*, which is consistent with previous reports (Jomoui et al., 2023; Wu et al., 2021; Zhang et al., 2025). Among 291 subjects with confirmed β-globin mutations, the most common genotype was *β* ^*CD17(A>T)*^ */β* ^*N*^, and there was no significant difference compared to previous reports (Song et al., 2025). The study in the southern region of Hainan found that α-globin mutations is mainly characterized by*-α* ^*4.2*^ */αα*, *-α* ^*3.7*^ */αα*, and *-α* ^*3.7*^ */-α* ^*4.2*^, while β-globin mutations is dominated by *β* ^*CD41-42(-TCTT)*^ */β* ^*N*^, accounting for 69.6% (Yu et al., 2022). The number of routine α-globin mutations in our study was more than that of routine β-globin mutations, and there was no significant difference compared to previous reports (Wu et al., 2021; Peng et al., 2020; Zhuang et al., 2020). Overall, the genotype distribution of thalassemia in our local population was generally consistent with previous reports, whereas the predominant genotypes varied considerably across different geographic regions, highlighting the necessity of taking regional genetic heterogeneity into full consideration in thalassemia prevention and control strategies (Dai et al., 2017; Falchetti et al., 2006; Lam et al., 2022).

Broadly speaking, standard hematologic profiling of MCV, MCHC, and MCH, when paired with hemoglobin electrophoresis, serves as an effective net for capturing the vast majority of thalassemia carriers; consequently, routine *α*/β-thalassemia genotyping acts as a confirmatory seal on the diagnosis. Yet, certain genetic variants remain elusive to these conventional analytical lenses, hiding like shadows in plain sight. Thus, for cases where suspicion lingers, deep gene sequencing becomes an indispensable compass to navigate around missed diagnoses, thereby establishing a solid bedrock for both genetic counseling and prenatal assessmen (Chui et al., 2006; Wei et al., 2025). Given the absence of curative therapies, bolstered carrier screening and prenatal genetic diagnostics stand as the paramount strategies for curtailing disease incidence, particularly among families with a hereditary predisposition (Chui et al., 2006; Bain et al., 2023).

Rare globin gene variants were detected in 10 subjects with confirmed globin gene mutations, including four with rare α-globin mutations, five with rare β-globin mutations, and one with both rare both α-and β-globin mutations. In this study, one new gene variants of α-thalassemia case was identified, case 4: HBA1:c.300 + 48C > G. It has not been reported in HbVar, HGMD, and PubMed databases. Importantly, the patient presented with low hemoglobin concentrations. The causal relationship between this novel HBA1 variant and the patient’s severe anemia remains unclear, as the patient retains three intact α-globin genes. Other potential genetic or acquired causes cannot be excluded, and further functional studies are warranted.

Recent studies have validated the utility of NGS in thalassemia screening across Chinese populations. In a large-scale investigation of 229,246 individuals from Ganzhou, Jiangxi Province, NGS identified 180 novel thalassemia variants (68 α- and 112 β-thalassemia variants), with a population frequency of 0.079%, and documented 21 complex genotypes that would have been entirely missed by traditional methods (Huang et al., 2025). Similarly, a study in Southwest China using NGS combined with fluorescence PCR melting curve analysis in 1,093 Han individuals of childbearing age reported a thalassemia carrier rate of 11.89%, identified one novel mutation (HBA2:c.166del) and six rare genotypes, and noted that conventional Gap-PCR plus PCR-RDB would have failed to detect 10 out of 130 carriers (7.7%) (Du et al., 2024). Collectively, these findings support that sequencing-based approaches enhance the detection of rare and novel variants, providing a more accurate genotype-phenotype characterization essential for genetic counseling and clinical management.

Because thalassemia is highly heterogeneous, it is essential to clearly map out how often it occurs, its genetic patterns, and its spread in Chengdu, China. Public education, population screening, and prenatal diagnosis should be thefocus for thalassemia prevention and control in Chengdu.

In conclusion, we analyzed the hematological characteristics and genotypes of subjects with confirmed globin gene mutations at Xindu District People’s Hospital of Chengdu, and through the Sanger sequencing, for the first time, confirmed α-globin gene variant HBA1:c.300 + 48C > G. Our findings complement existing data on rare alpha-thalassemia gene types. The results of this study also revealed gene deletions and variants in patients with thalassemia in Chengdu, providing valuable basic information for genetic counseling and prenatal diagnosis of thalassemia in Chengdu. However, the sample size of this study was regional, Serum ferritin and other iron biomarkers were not included in the present analysis, although they were available for the majority of participants, due to the retrospective study design. Future prospective studies should incorporate iron metabolic markers for comprehensive evaluation. Leading to certain inherent limitations that should be addressed in the future.

## Data Availability

The original contributions presented in the study are included in the article/supplementary material, further inquiries can be directed to the corresponding authors.
